# Supplementary material for: Are carbon emissions trading and green financial instruments synergistic? -Comprehensive quantitative research based on content analysis
Source: PLoS One. 2024 Mar 7;19(3):e0298601. doi: 10.1371/journal.pone.0298601 (PMC10919601; doi:10.1371/journal.pone.0298601)
Supplement: S1 Data — (ZIP) [file pone.0298601.s001.zip › Data/地级市Z_F工作报告.zip!/╣π╬≈/│τ╫≤╩╨/└┤╫╘╣1⁄2╓┌║┼í╛┬φ┐╦╩2╛▌═°í┐_2013.docx]

各位代表：

　　现在，我代表市人民政府向大会作政府工作报告，请予审议，同时请市政协委员和列席会议的同志提出意见。

　　一、 2012年工作回顾

　　2012年，是我市全面实施“十二五”规划、加快发展的重要一年。在自治区党委、政府和市委的正确领导下，全市各族人民深入学习贯彻党的十八大精神， 坚持“稳中求进”的主基调，积极应对外需疲软、内需收缩和经济下行压力增大等困难和挑战，突出抓好“四大建设”，着力推进“五个打造”，在“稳增长、促转 型、扩内需、惠民生、保稳定”方面下功夫，全市经济社会保持平稳较快发展的良好势头，开创了建设面向东盟开放合作的区域性新兴城市新局面。

　　（一）经济持续较快增长。初步统计，全市完成地区生产总值530.75亿元，比上年增长11.8%，连续9年实现两位数增长。三次产业结构由上年的 29.5:40.1:30.4调整优化为27:41:32。财政收入完成66亿元，同比增长14.5%，总量排全区第9位；工业总产值完成476.49亿 元，同比增长12.4%，工业对GDP增长贡献率达50.3%；规模以上工业增加值实现175.13亿元，同比增长18.8%，增速排全区第6位，创近年 来最好水平；外贸进出口总额71.3亿美元，同比增长40.6%，总额继续保持全区第1位；社会消费品零售总额84.37亿元，同比增长16.5%，增速 排全区第4位；城镇居民人均可支配收入19370元，同比增长12%；农民人均纯收入6263元，同比增长16.6%，增速排全区第4位，比全区平均水平 高255元，较好地完成自治区下达的各项经济指标任务，全市经济总体实现持续较快发展。

　　（二）南崇经济带建设全面推进。《南宁—新加坡经济走廊南宁—崇左经济带发展规划》获自治区政府批准实施，上升为全区发展战略。坚持边规划边建 设，用猛力、下猛药推进南崇经济带建设，八大重点园区建设初见成效。扶绥空港经济区纳入中国—东盟南宁空港经济区发展规划，园区框架全面拉开，太阳能利 用、电动车制造等一批项目投产，广西糖果休闲食品产业园开园建设，福建金冠、冠龙糖果项目入园启动。崇左工业区扩区建设取得阶段性成效，中泰（崇左）产业 园在第九届中国—东盟博览会上成功签约，其中首批入园项目中粮屯河一期、桂顺矿业一期已建成投产，崇左东亚糖厂搬迁项目、中铝稀土项目已启动，崇左工业区 发展成为我市首个百亿元产值园区。中国—东盟青年产业园、崇左（东盟）国际商贸物流园、凭祥边境经济合作区、凭祥综合保税区、中越凭祥—同登跨境经济合作 区以及各县域工业园区基础设施和项目建设稳步推进。全市新增3个自治区认证园区，国家和自治区认证园区达到7个。园区经济总量大幅增长，完成工业总产值 285亿元，占全市工业总产值的59.8%，比上年提高18个百分点。2012年经济带新开工项目830个，完成投资150亿元，同比增长70%。南崇经 济带乘势而起，逐步成为我市开放合作的大平台、产业集聚的加速器、经济发展的主阵地，开创了“陆路东盟”发展新局面！

　　（三）支柱特色产业扩大发展。由“白黑”支柱产业逐步向“白黑红绿”四大支柱特色产业发展，产业结构进一步优化，一批关联度高、支撑力强的产业 项目实现投产或开工，产业竞争力进一步增强。糖业方面，推进东亚、南华、湘桂、丰浩、中粮屯河等糖业集团发展循环经济项目，全年糖业循环产业产值 228.33亿元，同比增长18.3%，其中制糖业产值205.35亿元，同比增长15.8%。矿业方面，通过落实价格调节基金、生产奖励政策等措施，帮 助锰企业进一步恢复和扩大生产，全年锰业产值80.82亿元，同比增长3.9%。南国铜冶炼等项目前期工作加快推进。红木产业方面，规划建设1000亩红 木产业加工基地，已有12家红木企业入园建设。红木产业从单一的成品销售，逐步向原木进口、加工销售、文化旅游延伸拓展。生态旅游产业方面，生态文明建设 高效推进。颁布实施《崇左生态市建设规划（2010—2020年）》。超额完成主要污染物减排约束性指标任务。全面完成“绿满八桂”造林绿化工程任务，综 合排名全区第2位。珍贵树种种植“十百千万”示范工程走在全区前列，建立珍贵树种千亩连片种植基地10片、示范校园100个、示范村屯1000个，涉及农 户81245户，完成珍贵树种种植266.68万株。旅游开发取得新进展，“五个100公里”旅游线建设步伐加快，德天小镇、中越国际旅游合作区等一批旅 游项目扎实推进，江州-德天百里山水画廊、崇左—友谊关百里边关历史文化游、左江百里山水文化生态游等旅游线品牌逐步打响。全年接待旅游总人数 985.47万人次，同比增长22.2%；旅游总收入66.81亿元，同比增长28.1%。同时，实施“抓大壮小扶微”工程卓有成效，产业创新和企业自主 创新不断提升，全市规模以上工业企业由2011年末的124家发展到2012年末的136家，其中超十亿元工业企业由14家发展到15家，超亿元工业企业 由60家发展到67家。中信大锰成为崇左市首家广西强优工业企业，中信大锰、新锰集团、湘桂糖业被评为广西百强企业。非公有制经济快速发展，全年实现地区 生产总值272.96亿元，占全市地区生产总值的51.4%。市人民政府被中国轻工业联合会授予“十一五”轻工业特色区域和产业集群先进集体。

　　（四）投资拉动持续有力。全社会固定资产投资持续增长，2012年投资达532.15亿元，比上年增加117.09亿元，增长28.2%，连续 三年净增100亿元左右，投资对经济增长贡献率达65%以上，成为拉动增长的主要动力。全市共组织实施固定资产投资项目3580个，同比增长30%。其中 自治区层面统筹推进的重大项目22个，完成投资21.25亿元；超亿元重大项目118个，完成投资91.4亿元；500万元以上项目投资占全年投资的 75%。重大项目建设强力推进，沙钢锰业硅锰合金、新振锰品中低碳锰等项目开工建设，祥盛木业刨花板等项目竣工投产。招商引资工作成效显著，新签订国内招 商引资合同项目307个，合同投资总额330.74亿元；国内招商引资项目到位资金307.03亿元；全口径利用外资项目到位资金1.52亿美元，同比增 长26.8%，引进了一批辐射带动力强的大项目，进一步增强了发展后劲。

　　（五）城乡面貌明显改善。中心城区控制性详细规划覆盖面进一步扩大，中心城市基础设施不断完善，辐射带动能力进一步提高。城市建设三年大会战和 建市10周年献礼工程项目已全部开工建设，“四路四中心四星级饭店”建设进展顺利，工业大道全线贯通，友谊大道北段延长线加快建设；人民会堂竣工并投入使 用，文化艺术中心完成主体工程建设；崇左圣展酒店建成开业，崇左东盟国际大酒店、天湖大酒店即将竣工。崇左壮族博物馆开馆使用，“八大住宅区”首批住房竣 工交付，城市规划建设展示馆启动建设，留邕四所学校完成搬迁并顺利开学。城乡建设统筹稳步推进，县城及小城镇建设力度加大，全市城建类项目完成投资185 亿元，市政设施建设55亿元。村镇规划集中行动有效开展，编制乡镇规划13项，村庄规划783项。城乡风貌改造全面铺开，完成改造5674户。名镇名村建 设步伐加快，大新县硕龙镇特色旅游名镇、扶绥县东门镇特色工贸名镇、凭祥市平而村名村建设工作取得阶段性成效。小城镇建设“书记工程”强力推进，7个试点 镇的城镇公共基础设施及镇区绿化、亮化、美化等明显改善。城镇化水平比上年提高1.5个百分点，达33%。目前，我市中心城区新楼林立，框架进一步扩大， 城乡一体化统筹发展，城镇更靓丽，设施更完善，环境更宜居，崇左正以崭新的面貌迎接建市10周年的到来！

　　（六）现代农业创新发展。农林牧渔业总产值完成230.53亿元，增长5.4%。粮食生产稳定发展，粮食播种面积完成181.52万亩，总产量 48.75万吨。蔗糖产业“4331”工程加快推进，甘蔗种植面积423万亩，在全市推行“小块并大块”土地流转16万亩，实施甘蔗高效节水灌溉工程 21.6万亩，形成了“江州模式”（专业化公司运作）、“龙州模式”（公司+基地+农户）、“扶绥模式”（制糖企业+协会+农户）三种经营管理模式，成为 广西现代农业发展的先行者，全国和全区多个现场会在崇左召开学习推广我市经验。预计2012/2013年榨季入厂原料蔗1900万吨、产糖236万吨，分 别同比增加156万吨和22万吨。特色种养产业加大开发，“菜篮子”工程建设进一步加强，蔬菜产量达81.21万吨，完成农业秋冬产业开发160万亩、农 作物间套种推广181.3万亩，完成林下经济产值21.1亿元。庭院龟鳖、山羊圈养、圈舍养蛇、珍珠鸭等优势特色养殖较快发展，江州区四坝屯淡水鱼养殖协 会入围自治区“菜篮子”水产品生产基地，获农业部水产健康养殖示范场资格。农业科技创新不断进步，良种化、测土配方施肥、水肥一体化、植保综合防控等技术 得到普遍应用。农业产业化发展水平不断提高，规模以上农业产业化龙头企业达77家，农民专业合作社累计达229户。左江治旱工程前期工作取得重大进展，驮 英水库项目建议书及灌区规划通过水利部审查；全市实现中央财政小型农田水利重点县或专项县建设全覆盖，冬春水利、病险水库除险加固、“五小水利”工程等水 利建设扎实推进。江州区被水利部授予“全国农田水利基本建设先进单位”。农业机械化稳步提高，“万亩甘蔗全程机械化示范区”辐射带动作用突显，全市耕种收 综合机械化水平达41.8%，比上年增长4.6个百分点。崇左农业正由传统农业向现代农业迈进！

　　（七）开放合作再创佳绩。大力实施"陆路东盟崇左看"战略，扩大外贸发展规模，外贸进出口总额连续4年排在全区第1位，出口总额连续6年排在全 区第1位。区域交流与合作深化发展，积极参与"南北钦防玉崇百"区域经济一体化发展；与泰国莫拉限府友城关系进一步加强，与越南谅山市建立友城关系，与老 挝他曲市签订建立友城关系意向书，与北京市西城区缔结友好区市关系，友城工作成为我市对外交流合作的重要渠道。开放合作基础不断夯实，崇左-钦州高速公路 实现通车，形成崇左"通边达海"一小时交通圈格局；崇左-靖西高速公路建设稳步推进，崇左-河内、崇左-高平国际道路运输线实现开通；边境口岸、互市点基 础设施不断完善。开放合作形式不断丰富，积极组织参加第九届中国-东盟博览会，成功举办第二届陆路东盟国际商务文化（壮族歌坡）节、走进东盟（北京）推介 会以及第六届中国（崇左）德天边关国际旅游节、第20届中越（凭祥）商品交易会、崇左（宁明）国际花山文化节等节会，进一步提升了崇左知名度。

　　（八）财税金融工作持续增进。在糖、锰、水泥等主要工业品市场低迷的严峻形势下，着力挖掘增收潜力，加强征管，同时努力培育新财源，大力发展商 贸物流第三产业，推动全市财政收入迎难而上、稳步增长，全年完成收入66亿元。其中财政部门组织收入17.1亿元，国税部门组织收入25.51亿元，地税 部门组织收入23.39亿元。县域经济蓬勃发展，财政收入持续增长，扶绥县完成收入14.18亿元，大新县10亿元，天等县3.6亿元，宁明县7.76亿 元，龙州县6.61亿元，凭祥市7.4亿元，江州区9亿元。扶绥、大新、宁明、凭祥、龙州五县（市）均实现1亿元以上的增收。在全区财政收入超过10亿元 的16个县（市）中，扶绥县和大新县名列其中。公共财政预算支出累计131.01亿元，增支27.91亿元，同比增长27.1%。金融机构优化升级，工商 银行崇左支行成功升格为二级分行，农行崇左分行实现搬迁，建行增设大新支行，金融大厦启动建设，全市成功组建5家小额贷款公司，25家企业被确定为上市后 备企业。各金融机构人民币各项存款余额437.41亿元，比年初增加67.42亿元，增速排全区第3位；各项贷款余额239.59亿元，比年初增加 36.64亿元，增速排全区第5位，金融服务能力和服务成效明显提升。筹融资渠道进一步拓宽，城投、建卓、建投、交投、旅投、矿投公司投融资平台日益发挥 作用。

　　（九）社会事业全面进步。10项为民办实事工程年度目标任务基本完成。新一轮扶贫开发攻坚战、兴边富民行动大会战全面开展，编制完成滇桂黔石漠 化崇左片区发展规划、207个贫困村"十二五"整村推进规划。科技创新能力明显提升，新增高新技术企业1家，培育各类创新主体13家、研发中心4家，全市 发明专利申请量66件。教育事业加快发展，义务教育均衡发展试点工作稳步推进，广西民族师范学院附属三所学校进入提质创优阶段，城南新区完成从幼儿园到大 学的教育全覆盖。文化体育事业成果丰硕，花山岩画文化景观再次列入中国世界文化遗产预备名单，保护、开发和利用工作进一步推进；全市47个村级公共服务中 心项目建设提前竣工，4个乡镇和85个村级篮球场全部建成。广播电视传播能力建设得到新提升，农村公益放映任务超额完成。医改任务全面完成，基层医疗卫生 机构运行机制不断完善，新型农村合作医疗农民参合率达98.55%。"五个民政建设年"深入推进，困难群众社会救助水平和标准不断提高，五保对象人均月供 养、城乡低保人均救助水平达到或高于自治区要求。诚信计生工作创新推进，人口自然增长保持稳定，出生人口性别比明显下降，实现国家、省级优质服务全覆盖。 就业局势保持稳定，城镇新增就业27230人，城镇登记失业率2.6%，低于自治区控制数1.6个百分点，农村劳动力转移就业新增74741人。城乡居民 养老保险实现全覆盖。人才强市工作有新进展，市级人才小高地增至5个。保障性安居工程建设和农村危房改造如期完成。华侨农林场体制改革工作稳步推进。物价 总体水平基本稳定，全年居民消费价格指数累计上涨3.1%，低于全区水平。社会和谐稳定模范市创建工作扎实推进，全市乡镇综治信访维稳中心全部实现规范化 建设。"驻村联户，拜师助民""驻村夜访""矛盾纠纷化解年"活动扎实有效。民族团结进步事业取得新进展。安全生产形势稳定向好，食品药品安全得到保障。 国防后备力量和民兵预备役建设、"双拥共建"活动深入开展。全市在全国"两会"、党的十八大期间实现了"五个不发生"和进京"零上访、零非访"目标，平安 建设品牌效应更加突显。

　　社会事业多项工作在全国全区领先，市外侨办被外交部授予"服务国家总体外交突出贡献奖"；市广电局被国家广电总局授予"全国广播电视村村通先进 集体"称号；市农机局被中国农业机械学会授予"全国农机科普工作先进集体"称号；我市率先在全区完成全市国有文艺院团改革和文化市场综合执法改革工作；全 市新闻上送采用率排在全区前列；基层老年协会规范化建设经验成为全国典型；社会公众安全感和维护社会稳定动态六项工作指标继续保持全区前列，政法干警满意 度排名全区第1位。

　　（十）政府自身建设不断加强。大力推进政府行政效能建设，积极开展"解放思想、赶超跨越"大讨论暨"阳光效能"主题活动和"行动学习"活动，政 府效能和政府执行力全面提升。民主法治建设和依法行政扎实开展。自觉接受人大监督，定期向人大汇报工作；加强与政协联系，向政协通报工作。全年共办理人大 代表建议63件、政协提案99件，办结率100%。行政审批工作不断加强，市政务服务中心综合办公楼落成启用，53个具有行政审批职能部门100%进驻， 行政审批效率不断提高。群众满意度稳步提升，2012年企业对崇左市投资环境满意度全区排名上升到第5位。坚决贯彻落实中央、自治区关于改进工作作风的" 八项规定"和"30条具体要求"，切实转变作风，密切联系群众。廉政建设扎实推进，行政监察和审计监督更加规范，政风、行风建设进一步加强。

　　一年来，人力资源、机构编制、统计、国有资产监督管理、法制、水库移民、供销合作、二轻、工商行政管理、质量技术监督、新闻出版、宗教、侨务、 涉台事务、机关事务管理、驻邕管理、防震减灾、无线电管理、气象、水文、人防、消防、边防、档案、信访、保密、国家安全、调处、老龄、红十字会、史志等工 作整体推进。供电、通信、邮政、海关、海事、出入境检验检疫、石油、烟草、盐业等行业和工会、共青团、妇联、科协、文联、侨联、残联、工商联、社科联等群 团组织在经济社会发展中发挥了重要作用。

　　全市经济社会呈现稳中求进、持续增长、投资强劲、消费活跃、外贸增长、民生改善、社会和谐稳定、人民安居乐业的良好局面，形势令人鼓舞，成绩可喜可贺！

　　一年来，面对复杂严峻的发展形势，全市各级各部门和各族人民团结一致、迎难而上、实干拼搏，在全面建成小康社会的大道上又迈出了坚实的一步。这 些成绩，来之不易，饱含着自治区党委、政府的关心与支持，展现了崇左人民"九牛拓荒、个个出力"的创业精神。这是市委正确领导、科学决策的结果，是市人民 代表大会及其常委会和人民政协监督支持的结果。在此，我代表市人民政府，向付出辛勤劳动的全市广大干部群众，向各位人大代表、政协委员，向各民主党派、工 商联、各人民团体、中直区直驻崇左各单位，向驻崇左人民解放军和武警部队官兵，向所有关心支持和帮助我市各项建设事业的各界人士，表示衷心的感谢和崇高的 敬意！

　　各位代表，回顾一年来的工作，我们深切体会到：必须坚持把"稳增长"作为经济工作的首要任务；必须坚持把扩大投资作为推动经济增长的主要动力； 必须坚持把加快南崇经济带建设作为工作主题；必须坚持把发展壮大"白黑红绿"四大产业作为优化产业结构的主要方向；必须坚持以群众满意作为全部工作的出发 点和落脚点；必须坚持"实干就是能力，落实才是水平""只为成功想办法，不为失败找理由"的理念，把各项工作落到实处。

　　在充分肯定成绩的同时，我们也清醒认识到，当前我市仍然面临一些困难和问题，主要表现在：经济总量偏小，基础薄弱，增长压力大；产业结构单一， 产业链条短小，市场竞争力不强；工业增速放缓，经济效益下降，财税后劲乏力；贫困面较大，农民持续增收难度加大，统筹城乡协调发展任务艰巨；开放意识有待 进一步提高等。对于这些问题，我们必须引起高度重视，采取措施，加以解决，把

　　全市改革发展各项事业继续推向前进。二、2013年目标任务和主要工作部署

　　今年是全面贯彻落实党的十八大精神的开局之年，是实施"十二五"规划承前启后的关键之年，也是崇左建市10周年的喜庆之年，做好今年工作意义深 远、责任重大。2013年政府工作的总体要求是：全面贯彻落实党的十八大精神，坚持以邓小平理论、"三个代表"重要思想、科学发展观为指导，围绕加快建设 面向东盟开放合作的区域性新兴城市、实现"富民强市"新跨越的战略任务，继续把握"稳中求进"的总基调，以加快转变经济发展方式为主线，以提高经济增长质 量和效益为中心，以加力开放合作、加快南崇经济带建设为主题，坚定不移、奋发进取、真抓实干，全力推进"四大建设""五个打造"，努力实现全市经济持续健 康发展、社会和谐稳定，以优异成绩迎接建市10周年，为与全国、全区同步全面建成小康社会打下良好基础。

　　2013年政府工作主要预期目标是：地区生产总值增长13%；财政收入增长12%；全社会固定资产投资增长22%；规模以上工业增加值增长 18%；社会消费品零售总额增长17%；外贸进出口总额增长18%；城镇居民人均可支配收入增长13%；农民人均纯收入增长15%；居民消费价格指数涨幅 控制在4%以内；城镇登记失业率控制在4.2%以内；人口自然增长率控制在8.8‰以内；节能减排指标按照自治区下达任务完成。

　　为实现上述目标，我们将重点抓好以下八个方面的工作。

　　（一）加力开放合作，加快南崇经济带建设

　　更加全面深入地解放思想。把解放思想作为落实"两加"工作的首要任务，坚持"以空间换时间、以资源换产业、以存量换增量"，以园区建设为支撑， 以产业转型升级为动力，以更大的勇气和智慧推进"两加"工作。树立"非禁即可"的理念，用好用足用活政策，争取上级更大的支持，研究出台更加优惠的政策， 为开放合作创造更多的发展条件。解放思想不能仅停留在思想层面，更要在具体行动中体现，在落实工作中体现，在推动发展中体现！

　　积极参与区域合作。在加强与国内友城合作的同时，进一步扩大国际友城合作，探索全方位、多层次、宽领域的合作机制，促进区域交流与合作。深化与 泰国莫拉限府、越南谅山市友城合作关系，推进年内与老挝他曲市、缅甸勃固市、柬埔寨腊达那基里省签订友城协议。主动融入"南北钦防玉崇百"区域经济一体化 发展；积极参与桂港、桂台经贸合作以及珠三角、长三角等多区域合作；积极推动中越"两廊一圈"、南新经济走廊、中国-东盟自贸区建设。

　　加快推进与东盟国家互联互通建设。加快推进凭祥边境经济合作区规划调整方案的批准实施；开展中越水口-驮隆跨境经济合作区前期工作；全力推动凭祥沿边开发开放试验区报批工作。抓好边境口岸、互市点建设，加快推进爱店、硕龙口岸升格工作，大力推进14个边民互市点建设。

　　大力推进重点产业园区建设。继续组织实施南崇经济带规划建设，加快八大重点园区基础设施和产业项目建设。扶绥空港经济区全力推进扶绥至吴圩机场一级公 路建设，抓好广西糖果休闲食品产业园项目建设，大力发展空港经济。中国-东盟青年产业园继续推进南国铜冶炼项目，力争年内实现开工。

　　崇左工业区全面完成工业大道建设任务，加快崇左东亚糖业循环综合利用等项目建设，谋划铝材深加工项目入园，推动中泰（崇左）产业园上升为两国政 府层面的产业园区。崇左（东盟）国际商贸物流园加快中国-东盟特色产品大市场建设，力争商超中心项目年内实现开业。凭祥边境经济合作区抓好红木产业加工基 地等项目建设。凭祥万通物流园重点建设红木原木及半成品交易中心。凭祥综合保税区实现保税物流加工项目入园建设，加快保税区规划调整。中越凭祥-同登跨境 经济合作区继续加快推进规划建设工作。同时，推动县域园区升级工作，进一步做强做大县域经济。力争全市园区工业总产值完成360亿元，工业项目投资完成 107亿元，基础设施投资完成13亿元以上。

　　强力推进产业转型升级。以"白黑红绿"四大产业为重点，在精深加工、延长产业链、提高附加值上下功夫，促进产业转型升级。糖业方面，在扩大原糖产能的 基础上，大力发展精制糖、阿拉伯糖、蔗糖脂、木糖醇、朗姆酒以及果糖系列产品等一批循环及深加工项目。力促中粮屯河甘蔗循环经济项目二期今年动工建设，推 进宁明东亚生物质能发电、宁明丰浩甘蔗高效循环经济等项目开工建设，加快福建金冠、冠龙糖果项目建设，力争湘桂糖业酵母抽提物、凭祥丰浩生物肥循环经济等 项目竣工投产。力争推动崇左糖业循环基地建设上升到国家层面，筹划设立以食糖交易为主的广西崇左国际商品交易所。锰业方面，进一步扩大锰矿开发力度。鼓励 企业自主创新，研发生产高附加值的锰系产品，推进中信大锰锰系新材料深加工、新锰集团中低碳锰铁合金、珀琳锰资源深加工循环利用等项目建设，实现中信大锰 年产6万吨电解金属锰、沙钢锰业硅锰合金技改扩建项目竣工投产。有色金属和建材业方面，大力推进中铝稀土产业园建设，力争开工建设稀土分离生产线、龙州氧 化铝加工项目。加快推进南方水泥、红狮水泥等建材项目建设。红木加工业方面，加快推进红木加工项目建设，拉长红木产业链。同时，积极培育发展高端食品加 工、新材料、新能源等战略性新兴产业，进一步增强经济发展实力和后劲。

　　做强做大边境贸易。进一步扩大外贸进出口规模，促进各种贸易方式持续稳定增长。推进与东盟的经贸合作，加快以贸易为主向贸易与投资合作并重转变，以商 品贸易为主向加工贸易、技术贸易、服务贸易协调发展转变。支持和引导企业做好纺织品、机电产品、农副产品等传统大宗商品出口；想方设法扩大进口，多进口与 我市产业对接的产品，促进对外贸易平衡发展。大力发展边境加工业，推动凭祥发展红木、摩汽配件和农副产品加工，龙州发展农副产品进口加工，宁明发展中草药 出口加工、仓储物流及商贸服务等产业。

　　实施创新驱动发展战略。积极推进科技体制创新，提高自主创新能力。进一步深化与自治区科技厅的厅市会商机制。继续加强糖业、锰业等优势产业科技攻关。 推进崇左市科技创业园建设。鼓励企业创新发展，重点建设一批产业创新基地、平台、中心。今年建设45个、竣工投产11个亿元以上重点技改项目。加大专利工 作力度，提高发明专利申请量和拥有量。深入实施"质量兴市"战略，加大质检中心建设力度，加强产品质量安全监督保障。

　　（二）实施农民收入倍增计划，推进"三农"工作增加农民家庭经营收入。积极推进农业科技创新，加快发展现代农业，促进农民增收。稳定粮食生产，完成播 种面积180万亩以上，总产量保持在48.5万吨以上；加强粮食仓储设施建设，确保粮食安全。推进蔗糖产业"4331"工程，加快甘蔗高产示范和良种繁育 基地建设，力争完成甘蔗高效节水灌溉项目30万亩，甘蔗良种率继续保持100%。扶持发展水果、有机茶、指天椒、剑麻、八角等优势特色产业。实施"菜篮 子"工程，加大农业秋冬产业开发力度。建设农作物间套种技术集成示范带，面积稳定在180万亩以上。大力发展养殖业，稳定生猪生产，发展家禽养殖和特色养 殖，完成水产品总产量6.39万吨，肉类总产量12.52万吨。大力发展林下经济，实现产值27亿元以上。大力发展观光农业、休闲农业、"农家乐"，推进 农业向"接二（产）连三（产）"延伸，带动农民增加收入。

　　增加农民工资性收入。加快农村劳动力转移就业，新增5.6万人，实现累计在外稳定转移就业达50万人以上，实现劳务经济总收入60亿元以上。实施职业 培训扶贫攻坚工程，提高农民就业创业能力。开展就业服务指导，鼓励农民就近就业及外出就业农民"回乡"创业。科学布局劳务输出走向，开展区域劳务合作。鼓 励边民参与互市贸易，增加边民收入。

　　增加农民转移性收入。严格落实种粮直补、良种补贴、农资综合补贴、农机具购置补贴等各项惠农政策，确保各项补贴资金及时足额兑现到农民手中，直接增加 农民收入。加大对专业大户、家庭农场、农民合作社等新型生产经营主体的补贴力度。争取国家出台糖料蔗良种补贴政策。落实0-3公里边民生活补助政策，落实 农村低保对象月人均补助增加10元。继续实施农村危房改造工程，6月底前完成8050户改造任务。

　　增加农民财产性收入。按照依法自愿原则，引导农村土地承包经营权以转包、互换、转让、股份合作等形式有序流转，重点推广"小块并大块、分钱不分田"模 式。鼓励和支持承包土地向专业大户、家庭农场、农民合作社流转，发展多种形式的适度规模经营，让更多农民获得财产性收入。提高农民在土地增值收益中的分配 比例。

　　加强农业基础建设。实施基本农田整治和中低产田改造等项目。加强农田水利建设，实施灌区末级渠系和"五小水利"工程建设。如期完成自治区下达年度 10.32亿元冬修投资任务。投资6848万元建设农村饮水安全工程250处，解决10万农村居民及2万中小学师生饮水安全问题。强化动植物疫病灾害、农 业气象灾害等防灾减灾基础设施建设，抓好重大动物疫病防控。抓好农业机械装备建设，提升农业机械化水平。

　　进一步深化农村改革。推动城镇化和农业现代化协调发展，深化华侨农林场体制改革，加快集体林权制度配套改革，增强农村发展活力。鼓励和扶持农民兴办专 业合作和股份合作等多元化多类型合作社。推动农超对接、农校对接、农企对接等多种形式的产销衔接。引导金融机构加大对农村地区的信贷投入和保险支持力度。 抓好政策性甘蔗种植保险试点工作。进一步健全基层农技、水利等服务体系。

　　（三）实施城镇居民收入倍增计划，提高城镇居民生活水平

　　稳步提高职工工资收入。健全企业工资分配机制，推动企业开展工资集体协商和集体合同制度。加快完善企业工资正常增长机制。今年再调整最低工资标准，增 长20%以上。加强执行最低工资标准的检查监督，确保最低工资保障制度的落实。推进事业单位收入分配制度改革，做好事业单位实施绩效工资发放工作，统筹考 虑事业单位退休人员待遇，提高事业单位职工工资水平。

　　提高城镇最低生活保障标准。提高低保标准和补助水平，对符合条件的困难群众实行应保尽保，城镇低保对象月人均补助增加20元。

　　继续实施"全民创业"。加大创业扶持，落实创业税收优惠、担保贷款、资金补贴等政策。积极鼓励各行业放开创业，培育壮大创业主体，扶持发展微型企业，支持就近、就地创业，拓宽增收渠道。力争实现城镇居民经营净收入人均4000元以上，同比增长15.9%以上。

　　实现更高质量的就业。继续实施积极的就业政策，促进城镇劳动者充分就业，消除"零就业家庭"，实现劳动报酬稳步增长。加强公共就业服务体系建设，健全 公共就业服务经费保障机制。支持城镇发展食品加工、建筑业、物流业、运输业、旅游业和住宿餐饮等劳动密集型产业，创造更多的就业岗位。紧贴重点投资项目用 工需求，引导居民合理就业。实现城镇新增就业2万人以上。

　　（四）加快城镇化建设，推进城乡一体化发展

　　完善城乡规划编制。构建科学合理的城乡布局。坚持城市新区与产业园区互动发展，坚持新区建设与旧城改造相结合。加快城市基础设施、生态环境保护、公共 服务设施等方面的规划编制和实施。加快编制小城镇总体规划和村庄规划，打造特色小城镇集群和"一村一特色"新农村。规划要体现东盟、壮族、现代、生态等特 色。

　　推进中心城市建设。集中力量推进建市10周年献礼工程建设，确保今年8月6日前31个项目竣工投入使用。重点推进"四路四中心四星级饭店""八大住宅 区"、市委党校迁建、城市规划建设展示馆、旧城区污水管网改造二期工程、丽江新区等项目建设，进一步完善中心城区基础设施。规划开发水口湖片区，主要抓好 水口湖片区修建性详细规划，推进水口湖整治，加快龙峡山西路延长线、石景林西路延长线、连城路等路网建设，加快推进崇左大桥项目前期工作。推动中心城区与 崇左工业区联动建设，加快连接两区的路网建设，加快开发科教片区。继续开展园林城市创建活动。

　　推进县城和小城镇建设。继续推进和完善扩权强县工作，抓好扶绥县东门镇、凭祥市夏石镇的扩权强镇试点工作。加快推进撤乡改镇和撤镇改街道工作。加大城 镇建设投入，推进县城和中心镇的道路、供电、供水、供气、学校、医院、文化体育场馆、生活垃圾和污水处理等公共服务设施建设。继续实施小城镇建设"书记工 程"，7个试点镇在完成"十个标准"建设的基础上，推行城乡统筹发展试点，提升"书记工程"成效。加快推进全区试点名镇、名村建设。力争今年底全市城镇化 水平达35%以上，新增城镇人口5万以上。

　　推进工业化与城镇化互动发展。按照中央关于"促进工业化、信息化、城镇化、农业现代化同步发展"要求，以增强城镇承载能力支撑产业发展，以产业增长促 进城镇扩张，推进产业与城镇资源的有效整合，统筹推进新型工业化和城镇化协调发展。推进"抓大壮小扶微"工程，培育发展工业企业，为促进工业化与城镇化的 良性互动提供产业支撑。力争百亿元企业培育有新突破，确保十亿元、亿元企业数量稳步增加，新增一批上规模企业和1000家微型企业，扶持发展300家创意 机构。

　　推进户籍制度改革。坚持自愿原则，为失地农民、无土安置移民及进城创业、务工农民办理城镇居民户籍手续，享受城镇居民的社会保障和福利待遇。引导有条件的农村居民就近向城镇转移落户，有序推进农村居民转为城镇居民。

　　（五）进一步扩大内需，促进投资和消费较快增长

　　强力推进重大项目建设。继续推行"四个非常"和"六个一"工作机制，做到前期项目抓开工，开工项目抓进度，在建项目抓竣工。加快项目建设进度，重点推 进自治区和市级层面统筹、城市建设三年大会战、建市10周年献礼工程等项目建设。强化项目保障服务工作，抓好征地拆迁、城乡建设用地增减挂钩、"征转分 离"工作，争取更多用地计划指标。交通建设方面，加快推进南宁至凭祥铁路扩能改造和崇左至水口、隆安至硕龙高速公路、大新至科甲二级公路等项目前期工作。 推进崇左至靖西高速公路和龙州至科甲、坛洛至崇左、天等至德保、隆安经进结至天等、驮卢至大新等二级公路建设。大力推进新环、濑湍、将军岭作业区和左江崇 左至南宁Ⅲ级航道工程等项目建设。水利建设方面，继续推进左江治旱工程项目前期工作，力争驮英水库工程年内获国家立项。完成56座小型病险水库除险加固工 程建设任务。抓好中小河流综合治理工程、中央财政小型农田水利重点县建设。能源建设方面，推进崇左工业区热电联产项目前期工作，推进宁明诺沃奇项目二期、 大新下雷水电站项目建设。引导和鼓励有条件的企业发展自备电厂，缓解电力供应紧张问题。继续推进电网主网架和变电站工程建设。开工建设天然气管道工程项 目。产业项目方面，加大实体经济投资力度，加快实施一批投资强度大、拉动作用强、技术含量高、市场前景广的重点产业项目，推动产业集群发展。

　　继续做好财税金融工作。继续深化财税管理制度改革。落实积极财政政策，加强财源建设，强化税收征管。优化财政支出结构，加大民生领域投入，加大对"白 黑红绿"四大产业、工业园区及自主创新的扶持力度。积极争取上级财政资金扶持。严格控制一般性支出，不断提高政府预算透明度。加大筹融资力度，成立市筹融 资办公室，逐步组建县级金融工作办公室，指导开展筹融资工作。充分发挥城投、建卓、建投、交投、旅投、矿投公司等投融资平台作用，整合资产，增强筹融资能 力。完善国有资产管理体制，抓好留邕资产盘活开发。筹建崇左市中小企业服务中心，建立市、县联动的中小企业融资担保体系，为中小企业融资、项目投资做好服 务。加快培育上市后备企业资源，推进企业上市融资和再融资。继续深化政银企合作。加大金融招商力度，引进域外金融机构在我市设立分支机构。进一步落实财 税、金融等政策，鼓励、支持、引导非公有制经济发展。推行BT、BOT等市场化融资方式，鼓励民间资本参与投资社会事业。

　　加大招商引资力度。敢于让大利招大商引强商，坚持招龙头引强企。按照南崇经济带产业规划和园区规划，重点抓好面向香港、澳门、广东的招商引资，积极引 入产业链龙头企业、支柱型大项目，力争在引进世界500强、全国500强以及央企、知名民企、港澳台企业上实现新突破，确保今年招商引资实际到位资金 320亿元以上，利用外资1.6亿美元以上。

　　努力扩大消费需求。引导居民在崇左消费。推动消费结构升级，改善人民群众的生活质量。推动节假日消费和展会消费，积极扩大消费需求，促进城乡消费。促进房地产市场平稳健康发展。继续加强物价调控，稳定物价总体水平。力争今年全市社会消费品零售总额突破100亿元大关。

　　大力发展物流业。培育和发展壮大一批物流企业，提升商贸物流专业化和现代化水平。加快推进崇左（东盟）国际商贸物流园、凭祥综合保税区物流体系、中国 -东盟（凭祥）农副产品专业市场、中越边境中草药材商贸物流基地、中国-东盟（宁明）国际农产品综合批发市场、龙州东盟国际商贸城等项目建设。把崇左打造 成为南新经济走廊上重要的物流节点城市和中国-东盟开放合作物流基地。

　　推进文化旅游产业。实施"快乐崇左"工程，提升崇左"吃、住、行、游、购、娱"档次，促进文化与旅游深度融合发展，打造"边关旅游胜地"。大力发展住 宿餐饮业，推进宾馆上星工作，引进特色餐饮，打造崇左美食品牌。开发特色旅游商品，建设一批旅游商品市场，力争建立免税购物区，引进东南亚等异国风情演 艺，拉动旅游消费。组织开展面向东盟的跨国游，推进与东盟国家的互通互游。加快整合开发全市旅游资源，完成《崇左市旅游发展总体规划》《花山风景名胜区规 划》修编。加快建设"五个100公里"旅游线，做好景区创A工作，深度开发建设石景林、龙峡山森林公园、乐养城、龙谷湾、桃花岛、花山岩画、弄岗、上金旅 游区、明仕田园、德天瀑布、恩城风光、派替湖、丽川公园、友谊关、红木文化商城、大连城等景区景点。推进左江旅游码头、中国-东盟（崇左）旅游集散中心等 项目建设，推进桂台文化旅游产业园、壮族风情园等项目前期工作。大力推进德天小镇和德天景区"中越一条街"建设，加快中越友谊关和德天o板约瀑布国际旅游 合作区发展。完善旅游服务配套设施，建好旅游信息咨询服务中心。

　　（六）加快生态文明建设，努力建设美丽崇左

　　组织实施主体功能区规划。按照《广西主体功能区规划》的具体划分，江州区、凭祥市属于自治区层面重点开发区域，天等县属于限制开发区域的重点生态功能 区，扶绥县、大新县、宁明县、龙州县属于限制开发区域的农产品主产区。各县（市、区）要严格按照主体功能区规划定位，合理安排生产、生活和生态空间，进一 步优化我市城镇化格局、农业发展格局、生态安全格局。加大对花山风景名胜区、白头叶猴保护区、弄岗自然保护区、恩城自然保护区、左江母亲河等生态资源的保 护和利用。

　　切实抓好节能减排。加大落后产能淘汰力度，抓好重点耗能行业和企业节能降耗。认真落实总量减排目标责任。继续抓好城市、城镇污水管网延伸及改造，加强污水处理厂运营管理。加快推进农业源污染减排和机动车减排工作。

　　推进资源节约和综合利用。保护和节约耕地资源，积极探索土地节约集约利用新机制，继续推进国土资源节约集约模范县创建活动。提高水资源合理利用率。加 强矿产资源勘查、保护、合理开发。推行资源综合利用和清洁生产，引导制糖、建材、有色金属等企业发展循环经济。继续开展节能高效照明产品推广应用工作。

　　大力实施植树造林活动。在完善提升"绿满八桂"崇左造林绿化、珍贵树种种植"十百千万"示范工程的基础上，重点实施珍贵树种种植"三个一万"工程，在 市、县城区街道种植1万株大苗、主要公路沿线及山坡种植1万亩、国有林场种植1万亩珍贵树。积极申报国家珍贵树种培育示范市，推进"森林城市"创建工作。 抓好水土保持重点工程建设。抓好天然林、公益林保护，实施退耕还林、沿江防护林建设，推进石漠化治理。

　　进一步加强环境保护。积极推进生态文明示范市建设。开展PM2.5监测工作。继续开展环境风险和安全隐患大排查大整改，建立健全环境风险防范机制和环 境突发事件应急处理机制，成立崇左市环境应急与事故调查中心，妥善处置各类突发环境事件。加强主要河流水质的污染防控和集中式饮用水水源地保护工作。深入 开展环境综合整治，改善城乡环境。

　　（七）切实保障和改善民生，推动社会事业全面进步

　　深入推进新一轮扶贫开发。实施产业扶贫、科技扶贫、教育扶贫、生态扶贫和社会扶贫，鼓励和支持非公企业参与扶贫开发。推进滇桂黔石漠化片区区域发展与 扶贫攻坚工作。积极争取1亿元以上的中央和自治区扶贫专项资金，用于整村推进全市207个贫困村的扶贫开发，加强贫困村基础设施、产业开发、生态环境、社 会事业以及基层组织建设。启动兴边富民行动大会战，推进边境开发，建立健全兴边富民补贴补助机制。

　　优先发展教育事业。规范和提高各阶段学校管理与服务水平，深入实施素质教育，全面提高教学质量和义务教育巩固率。抓好学前教育，推进乡镇中心幼儿园建 设。巩固义务教育阶段学校常规管理和内涵发展工作成果。推进普通高中多样化和特色化发展。深化职业教育攻坚五年计划，加快国门职教园区建设。提升广西民族 师范学院三所附属学校办学水平，在城南新区新建1所小学，引进民间资本兴办1所幼儿园。加快推进五所院校整合转制和广西工贸高等专科学校筹建工作。支持我 市高校与东盟国家开展教育合作交流。完善和落实学生资助政策体系。

　　推进医疗卫生和人口计生事业。全面推进医改工作，巩固完善基本药物制度，加快基层医疗卫生服务体系建设，积极推进县级公立医院综合改革试点，不断健全 和完善医疗卫生服务体系。加强全民医疗保障制度建设，城镇职工、城镇居民和新农合等三项基本医疗保险参保率稳定在97%以上，城镇居民医保、新农合人均筹 资标准提高到340元。

　　加快推进市人民医院创建“三甲”工作。推进广西民族医院与广西医科大学协作共建，推进广西民族医院与市人民医院合作共建。力争市第二人民医院投入使 用，推进市儿童医院、市疾控中心等项目建设。加强食品药品安全监管，加强食品药品检测能力建设。做好诚信计生工作，加强出生人口性别比综合治理，实施免费 孕前优生健康检查，继续推进国优创建工作。

　　推进文化体育事业。加强社会主义核心价值体系建设，积极创建全国未成年人思想道德建设工作先进市。深化文化体制改革。加强公共文化基础设施建设，加快 建设市文化艺术中心，完善市、县、乡、村四级公共文化服务体系。继续实施公共博物馆、图书馆、文化馆（站）免费开放。实施文化信息资源共享、农村公益电影 放映、农家书屋等工程。加强壮族文化传承与挖掘，抓好文艺精品创作，设立文学艺术创作“花山奖”。深入开展“百团千场”群众文化活动，组织办好山歌比赛、 广场文艺演出等活动。推进广播影视事业发展，创办崇左网络广播电视台，增设崇左电台新频率，进一步提高广播电视传播能力。抓好左江花山岩画的保护、开发和 利用，全面启动花山岩画文化景观申遗工作，启动建设花山岩画博物馆。广泛开展全民健身活动，促进群众体育和竞技体育全面发展。加快市体育中心项目建设，举 办崇左市第二届运动会。

　　完善社会保障体系。继续完善城乡居民社会养老保险工作。完善被征地农民社会保险政策。提高各项社会保险待遇水平。强化社会保险基金监督管理。健全社会 福利机构，完善五保供养、社会救助制度，建立城乡低保分类施保机制。加强保障性住房建设和管理，继续推进驻邕危旧房改住房改造工作。发展老龄服务事业和产 业。推进残疾人事业和慈善事业，完成14个“残疾人扶贫基地”建设。

　　扎实推进人才强市战略。优化人才政策措施，引进和培养我市急需、紧缺的糖业、矿业、红木加工、旅游、外贸、物流等方面的人才。进一步加强人才小高地建设，继续组织开展人才服务基层活动。

　　坚持为民多办实事好事。按照自治区统筹安排，筹措资金29.08亿元，继续为民办好10件实事。一是社保惠民工程。筹措资金4.41亿元，实施城乡居民养 老保险、城乡居民最低生活保障、边境0—3公里边民生活补助、敬老院和五保村扩建维修、农村住房政策性保险等项目。二是健康惠民工程。筹措资金4.26亿 元，实施新型农村合作医疗、基本公共卫生服务、防治艾滋病攻坚工程、母婴健康“一免二补”幸福工程、地中海贫血防治等项目。三是教育惠民工程。筹措资金 4.36亿元，实施扩大农村学前教育资源、农村义务教育学生营养改善计划、农村义务教育学校建设、普通高中国家助学金、职业教育基础能力建设、学生资助等 项目。四是强基惠民工程。筹措资金5.18亿元，实施修建贫困村屯级路、小型农田水利重点县建设、农村安全饮水解困工程等项目。五是安居惠民工程。筹措资 金 5.16亿元，继续实施保障性住房、棚户区改造、廉租住房租赁补贴、农村危房改造、城乡风貌改造等项目。六是土地整理惠民工程。筹措资金 5840万元，同时争取上级专项资金补助，在 5个国家农业综合开发县（市、区）建设中低产田改造项目、高标准农田示范工程项目，在贫困地区实施坡改梯和基本农田建设。七是农补惠民工程。筹措资金 2.08亿元，实施农作物良种补贴、农资综合补贴、农机具购置补贴等项目。八是生态惠民工程。筹措资金1.18亿元，实施 “绿满八桂”工程、珍贵树种种植、石漠化综合治理、森林生态效益补偿、沼气池建设等项目。九是文化惠民工程。筹措资金2287万元，实施村级公共服务中 心、农民体育健身等项目。十是通畅惠民工程。筹措资金1.64亿元，组织实施自治区“千村公路通畅工程”，力争实现65个左右建制村建设沥青水泥路。

　　加强和创新社会管理。加强社会管理工作新机制、人才队伍和信息化建设。加强流动人口和特殊人群服务管理。加强舆情监测和舆论引导。深入开展社会矛盾纠 纷排查化解工作。健全和完善群众诉求表达机制、社会矛盾调处机制、重大决策制定的社会稳定风险评估机制等，从源头上预防和减少矛盾。严格落实安全生产责任 制，加强安全保障能力、应急救援体系建设。加强道路交通安全、消防安全、电力设施安全、环境安全工作。深入推进平安崇左建设，推进政法综治基层基础建设， 全面推进社会治安防控“八张网”和“八大员”建设。扎实开展严打整治专项行动，提高刑事侦查破案水平。深入推进法治崇左建设，严格执法、公正司法，加强法 制监督，进一步推进执法队伍建设。

　　巩固民族团结。深入开展民族团结进步教育，推进民族团结进步模范市建设。落实民族政策法规，保障少数民族合法权益。

　　加强国防建设。加强国防动员、国防后备力量建设和人民防空工作。推进双拥共建活动，巩固军地军民关系。加强边境管控，深入开展“爱民固边” 模范市创建活动，确保南疆国门安全稳定。

　　（八）加强政府自身建设，努力打造人民满意的政府

　　做好今年政府工作，必须以新的面貌、新的举措、新的作风、新的形象加强政府自身建设，努力打造公平正义、勇于创新、求真务实、廉洁高效的人民满意政府。

　　努力打造公平正义的法治政府。坚持依法行政、依法治市，把法治贯穿到政府决策、执行和监督全过程。加强法制教育，规范行政行为，做到依法行使职责。严 格执行人大及其常委会的决议决定，自觉接受人大法律监督和政协民主监督，认真办理人大代表和政协委员的议案、提案和建议，认真听取各民主党派、工商联、人 民团体和社会各界人士的意见。加大政务公开和政府信息公开力度，畅通政府与公众互动渠道，让人民群众更有效地监督政府。

　　努力打造勇于创新的服务政府。推进思想观念创新、体制创新、领导方式创新和工作机制创新，先行先试，敢闯敢干，用创新的理念推进工作，用创新的措施破 解难题，不断增强政府工作的灵活性和适应性。坚持“服务是政府的天职”理念，强化社会管理和公共服务职能。推进行政管理体制改革，加强政府绩效管理和考 评；深化行政审批制度改革，加强各级政务服务中心管理；建立健全公共资源交易市场，推动交易便捷化和公开公正。

　　努力打造求真务实的效能政府。坚决落实中央、自治区关于改进工作作风的“八项规定”和“30条具体要求”，改进调查研究，简化文风会风，改进新闻报 道，厉行勤俭节约，加强督促检查。加强协调、政令通畅，勇于负责、敢于担当，逢山开路、遇河架桥，切实提高政府执行力。把求真务实作为政府工作的根本要 求，多干打基础、利长远的工作，多做惠民生、促和谐的实事。巩固“阳光效能”活动成果，继续推行“一线工作法”，面对面解决群众问题，心贴心为民服务。以 求实效展现新作风，以高效率保证高质量，以抓落实推动大发展。

　　努力打造清正廉洁的阳光政府。严格执行党风廉政建设责任制和反腐倡廉各项规定，建立健全行政权力运行制约和监督机制，惩防并重，从源头上预防腐败。坚 决纠正损害群众利益的不正之风，着力解决人民群众反映强烈的问题，维护人民群众的合法权益。充分发挥监察、审计等专门监督的作用，主动接受人民群众监督和 新闻舆论监督，让人民监督权力，让权力在阳光下运行。

　　各位代表！回顾过去，辉煌成就鼓舞人心；展望未来，大好形势催人奋进。党的十八大已吹响全面建成小康社会的号角，让我们紧密团结在以习近平同志为总书 记的党中央周围，高举中国特色社会主义伟大旗帜，以党的十八大精神为强大动力，在市委的坚强领导下，紧紧依靠全市各族人民，解放思想，锐意进取，扎实工 作，努力建设面向东盟开放合作的区域性新兴城市、实现“富民强市”新跨越，向着与全国、全区同步全面建成小康社会的目标奋勇前进！
